# Bayesian Mixture Models for Assessment of Gene Differential Behaviour and Prediction of pCR through the Integration of Copy Number and Gene Expression Data

**DOI:** 10.1371/journal.pone.0068071

**Published:** 2013-07-12

**Authors:** Filippo Trentini, Yuan Ji, Takayuki Iwamoto, Yuan Qi, Lajos Pusztai, Peter Müller

**Affiliations:** 1 University Centre of Statistics in the Biomedical Sciences, Vita-Salute San Raffaele University, Milan, Italy; 2 Center for Clinical and Research Informatics, NorthShore University HealthSystem, Evanston, Illinois, United States of America; 3 Department of Breast and Endocrine Surgery, Okayama University Hospital, Okayama, Japan; 4 Division of Quantitative Sciences, MD Anderson Cancer Center, Houston, Texas, United States of America; 5 Chief of Breast Medical Oncology, Yale School of Medicine, New Haven, Connecticut, United States of America; 6 Department of Mathematics, University of Texas, Austin, Texas, United States of America; Cleveland Clinic Lerner Research Institute, United States of America

## Abstract

We consider modeling jointly microarray RNA expression and DNA copy number data. We propose Bayesian mixture models that define latent Gaussian probit scores for the DNA and RNA, and integrate between the two platforms via a regression of the RNA probit scores on the DNA probit scores. Such a regression conveniently allows us to include additional sample specific covariates such as biological conditions and clinical outcomes. The two developed methods are aimed respectively to make inference on differential behaviour of genes in patients showing different subtypes of breast cancer and to predict the pathological complete response (pCR) of patients borrowing strength across the genomic platforms. Posterior inference is carried out via MCMC simulations. We demonstrate the proposed methodology using a published data set consisting of 121 breast cancer patients.

## Introduction

### Biological Background

#### Copy number and arrayCGH

Human beings have two copies of each gene, defined as a segment of DNA. The *normal* copy number of a gene is therefore two. Copy number aberration (CNA) refers to cytogenetic events in which the DNA replication process is disrupted such that the gene either is replicated multiple times (copy number gains) or loses one or both copies (copy number loss) in newly generated cells. Comparative Genomic Hybridization (CGH) has emerged as a dominant technique for detecting CNA [1], especially when combined with microarrays. The resulting arrayCGH techniques [2], [3], [4] and [5] measure thousands or millions of genomic targets or “probes” that are spotted or printed on a glass surface. These probes usually span the whole genome with a resolution of the order ranging from 1 MB (one million base pairs) for BAC (bacterial artificial chromosome), to 50–100 kb (kilo base pairs) for more recent microarrays. In an arrayCGH experiment, a DNA *test* sample of interest is labeled with a dye (say Cy3) and then mixed with a diploid *reference* sample labeled with a different dye (say Cy5). The combined sample is then hybridized to the microarrays and intensities of both colors are measured through an imaging process. The quantity of interest is the 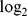 ratio of the two intensities for each color. The collection of the intensity ratios then provide useful information about genome-wide changes in copy numbers between the two samples. Since the reference sample is presumed to be diploid, the intensity ratio is determined by the copy number of the DNA in the test sample. If the copy number of the test sample is also two, then the theoretical 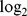 intensity ratio equals zero. If there is a single copy loss in the test sample, the theoretical ratio is 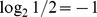 assuming all the cells in the test sample lost one copy of the DNA fragment. If there is a single copy gain, the theoretical ratio is 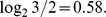 Multiple copy gains are called *amplifications*, and the corresponding theoretical intensity ratios are 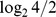, 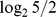, etc. When both copies are lost, the theoretical ratio is 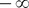 and a large negative value is usually observed in experiments.

#### Integration of DNA copy number and RNA expression

Expression microarrays measure RNA expression which, by the central dogma of molecular biology, are resulted from the transcription of DNAs. Microarray technology for measuring RNA gene expression has been well known to the statistical community, and its review is omitted here. Naturally, we are prone to think that CNAs impact the intensities of the relative RNA expressions in that more copies of DNA should lead to higher levels of RNA expression. It is therefore of great interest to study the intensity of such interaction, if there is any, between aCGH and RNA expression measurements on different genes.

Gene expression and copy number variation data have been broadly studied, to assess differential expression of genes [6] and to find segments along the DNA that show CNAs [7], [8]. Statistical and computational models for integrating different types of data are becoming a popular topic in the recent literature, even though only few considered full model-based approaches. [9] was among the earliest to investigate the direct association between the two types of data in breast cancer cell lines and tissue samples, and their approach was based mainly on descriptive statistics. Van Wieringen and Van de Wiel [10], attempting to mitigate the high noise in the raw expression measurements of the DNA and RNAs, proposed a sampling model for RNA expression incorporating estimated probabilities of corresponding CNAs. They subsequently developed nonparametric adaptive tests to study whether the estimated copy number variations in the DNA level would induce differential gene expression at the RNA level. More recently [11] presented a double-layered mixture model (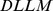) that directly modeled segmental patterns in the copy number data to produce CNA profiles, and simultaneously scored the association between copy number and gene expression data. The DLMM assigned high scores to elevated or reduced expression measurement only if the expression changes are observed consistently across samples with copy number aberration.

An important biological premise to the description of the model is that by integrating DNA copy number and RNA expression data, we will gain more knowledge about the underlying biological process. For example, a high or low correlation between a copy number aberration (CNA) for a gene marker and its abnormal RNA expression would indicate different carcinogenic mechanism and therefore different treatment selections [12] [13].

We describe a Bayesian Mixture Model that converts the noisy raw intensity measurement of the DNA and RNAs into probability of expression, which are subsequently modeled as latent parameters. Thus the integration of the two platforms is realized by joint modeling the probabilities of expression through a probit regression. Our aim, however, is not only to evaluate the relative contribution of large genetic variants such as CNAs, to gene expression but also make inference using both differential expression of the genes and differential copy number variations of the same set of genes. Moreover our full model-based approach allows us, after new information on the patients in the study are acquired, to exploit the latent integrated structure of our model and achieve better predictive performances for the clinical outcome of new patients coming into the study.

In the next paragraph we present a motivating example with matched arrayCGH and microarray samples from breast cancer patients. In the materials and methods section we introduce probability models with a particular focus on the probit regression that allows for integration of both platforms, along with some simulation studies. Thus, in the result section, the focus is on posterior inference of the interaction between the two platforms, *differential behaviour*, which takes into account both differential gene expression and differential CNA, and prediction of the pCR of patients after treatment. A final remark is provided in the discussion section.

## Motivating Example

We consider data in breast cancer consisting of 121 patients from three disease subgroups, ER+, HER2+, and triple negative (TN). ER+ patients have present estrogen receptors – a protein related to hormone and regulation of gene expression – in their cancer cells. HER2+ patients are instead those whose tumor cells test positive for a protein called human epidermal growth factor receptor 2. Finally TN patients lack three “receptors” in their cancer cells: ER, HER2, and progesterone receptors. ER+ and HER2+ patients were therefore collapsed in the same group, in order to compare TN patients versus others.

On a slightly reduced set of 116 patients we have a measure, formalized as a dichotomous variable, on their positive or negative pCR to treatment. Numerosities are specified in the table 1.

**Table 1:** Contingency table to classify patients with respect to subgroup of breast cancer and pathological complete response.

	Triple Negative	Positive to ER, HER2 or both	TOT
Positive pCR	20	11	31
No pCR	33	52	85
Missing	3	2	5
TOT	56	65	121

The mRNA expression data was obtained with Affymetrix U133A gene chips. The data was normalized with MAS5 algorithm, scaled to target intensity of 600 and log2 transformed. The expression profiles of the cancers are available at GEO accession number GSE22093 [14]. The DNA copy number data was generated with Agilent 4x44K CGH arrays, processed as log2 ratios of the intensities of the two colors, and is available at ArrayExpress accession number E-TABM-584.

ArrayCGH and microarray RNA experiments have been performed using the 121 breast samples to obtain the copy number data on 22,944 probes and RNA expression data for 11,306 genes. We then mapped 22,944 probes to the 11,306 genes, which gave us a matching between the probe ids on the aCGH and the gene ids on the microarrays.

## Materials and Methods

### Ethics Statement

All the research used public data, published in 2009 in the following paper: “Molecular characterization of breast cancer with high-resolution oligonucleotide comparative genomic hybridization array” written by Andre F. et al. and published in Clinical Cancer Research [14].

### Sampling model for 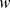 and 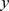


On arrayCGH, the experimental unit is probe 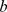 belonging to gene 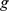. On RNA microarray, the experimental unit is gene 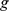. Denote 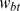 the log2 intensity ratio for probe *b* at sample *t*, and 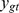 the RNA expression level for gene *g* at sample *t*, 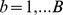 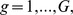 and 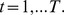 Denoting 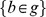 the set of arrayCGH probes corresponding to gene 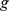, the matched copy number and RNA expression data for sample 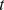 is then
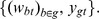
We propose mixture models for 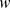 and 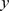 and introduce latent variables representing the differential expression status of the DNA and RNA, respectively. We then integrate the two models by constructing a prior probit regression linking the latent variables from both platforms.

We use a mixture model [15] to introduce trinary latent indicator variables for the CNA state for each probe and the differential expression (DE) state for each gene. Specifically, let 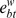 take values in the set 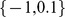, respectively corresponding to the copy-loss (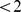 copy number), copy-neutral (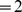 copy number), and copy-gain (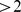 copy number) states and 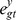 take values in the set 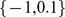, respectively corresponding to the under-, normal-, and over-expression states. Conditional on 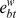 and 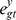, the sampling models for copy number log2 ratios 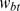 and for gene expression 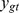 are given by
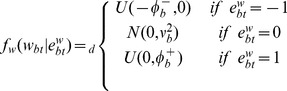
(1)

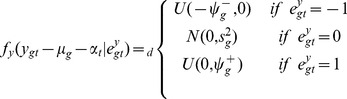
(2)In (2), the mixture model for gene expression data 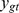 includes a gene effect 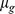 and a sample effect 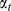. This is not the case in the mixture model for aCGH data 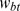. The main reason is because 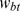 is already a log ratio between the cancer sample copy number and the reference sample copy number and therefore the corresponding effects should have canceled out by taking the ratio. The sampling model is indexed by 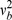 and 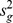 representing normal ranges of variability in the observed measurements 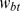 and 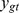. The parameters 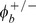 and 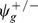 define the tail overdispersion with respect to normality, associated with copy losses or gains for aCGH and under- or over-expression for microarrays.

### Latent probit scores and probit regression

Anticipating the integration of both platforms using a regression model, we further introduce latent Gaussian variables 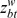 and 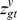 to define a probit scores for the trinary indicators 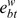 and 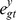. Specifically, define 
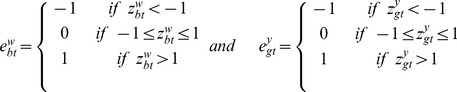
(3)


Before we introduce the probit regression for integration, we present a prior for 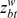 that allows for inference of different CNAs across different conditions, in our case of breast cancer data, different subtypes of breast cancer. Let 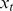 is a clinical categorical covariate indicating which subgroups the patient belongs to, we assume that
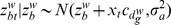
where 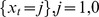 respectively if the patient belongs to TN subgroup or not, 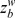, a probe-specific mean, describes a baseline CNA status (e.g., a reference subtype) and 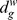 a trinary indicator accounting for differential CNA in the two subtypes, following a prior distribution given by



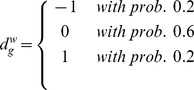



The integration of the two platforms is easily done using the latent probit scores and a linear model. First, we introduce a gene-level score for the aCGH data, defined as 
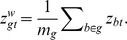
 Keeping in mind that there is a natural biological causal relationship between DNA copy number change and altered gene expression for the corresponding RNAs, we assume that
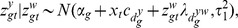
where 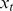 is the clinical binary covariate mentioned above, while 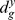 and 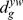 trinary indicators accounting respectively for differential gene expression in TN subgroup and interaction between the two measurement for gene 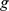, following similar prior to the one mentioned above for 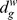.

#### Markov dependence across probes

A Markov dependence is assumed across the probes and it is defined in the following conditional prior on the probe specific effect. Define 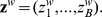 Assuming that the index 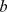 is ordered according to locus proximity on the chromosome, the dependence across adjacent probes is described as follows. Let 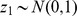 and
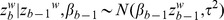
for 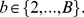 In this formulation the parameters 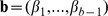 can be directly interpreted as partial correlation coefficients, defining the strength of dependence between 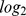 ratios associated with probes that are adjacent on the chromosome.

#### Priors

The last step is the specification of the priors for the set of parameters that index the sampling model. We assume conditionally conjugate priors. Denoting 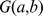 a gamma distribution with mean 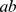, we assume
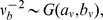


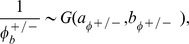


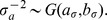



Particular attention is given to the formulation of the prior for 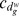 where
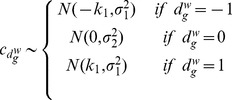
with 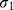 much larger than 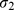 and 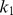 fixed at 1. The prior for 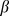
's is given by



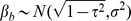
for 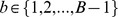, with 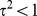 so that the marginal variance of 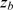
's is bounded above. Note that this model assumes that adjacent probes are equally correlated, characterized by 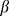
's and 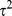. Alternatively, one could model the correlation between probes as a function of their genomics distances, and this can be easily achieved by modeling 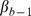 as a distance between probes 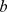 and 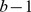, for example. Finally we assume conditionally conjugate priors for the gene and slide specific effects



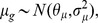


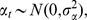
subject to 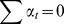. Finally, the normal range of variability in mRNA expression



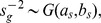



the tail over-dispersion parameters
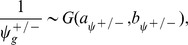
and the regression parameters



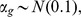


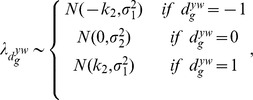


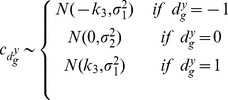
with the same assumptions on 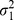, 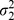 and 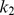, 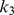 fixed at 1.

**Figure 1:**
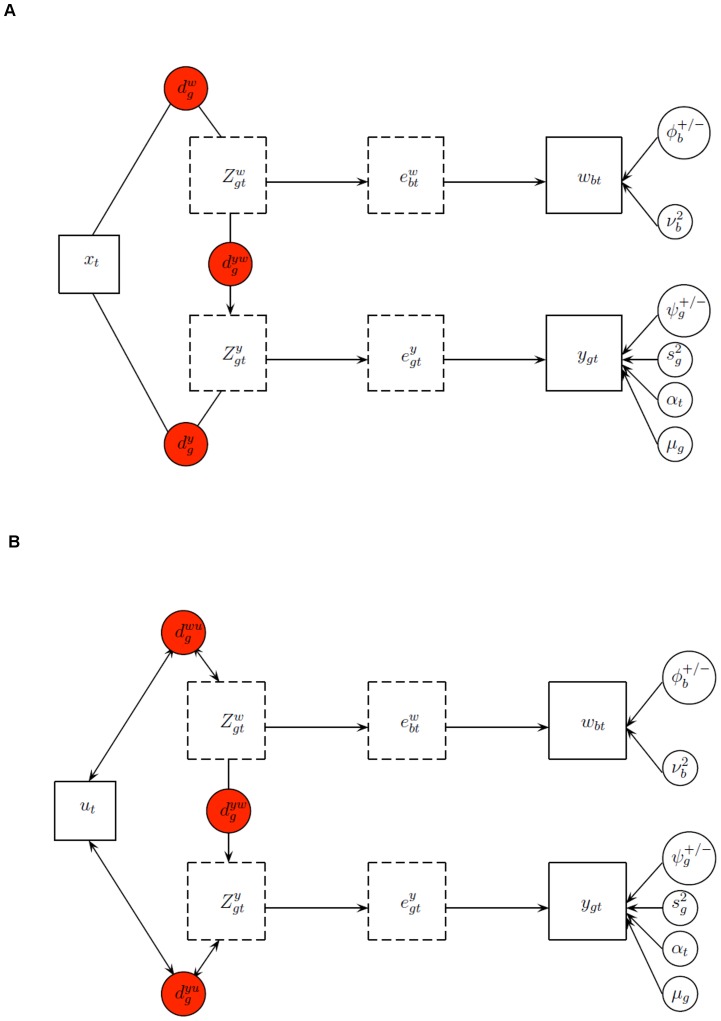
Graphical representation of the model for assessment of gene differential behaviour (A) and the prediction model (B). Boxes refer to variables in the model, where latent variables are represented by dotted line boxes. Circles refere to parameters, where the red ones are the indicators used for posterior inference.

A summary of the model is given in the upper part of Figure 1.

### Modified Probability Model for the prediction of pCR

The idea of this section raises from the question of whether or not we could use the same latent structure underneath gene expression and copy number variation data to make inference on a clinical outcome of new patients in the study, in particular 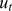, the pCR of patients to treatment.

The chosen approach is to state a model for 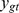 and 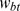, 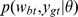, and to assume a Bernoulli distribution for 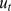. This leads us to the sought model 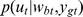 and posterior probabilities of 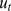 being 1 give us a measure for the prediction of the outcome of the new patient.

The advantages of our model with respect to, for example, a simple logistic regression 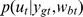 are mainly the noise reduction achieved through the assumption of a latent structure underneath our data, i.e. the latent POE scores for gene expression and the natural variable selection allowed within the model itself; indicators 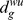 and 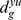 in equation (4) and (5) (with Bernoulli priors with probability 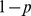 and 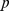 very close to 1) allow for a reduction of the number of covariates (genes) and avoid the problem of overestimation.

In summary, as a new patient comes into a study and we have measurements of his gene expression and copy number variation, we run the model 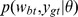 and assume for his clinical outcome 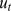 a Bernoulli distribution with probability 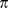. Through MCMC methods we obtain updated posterior probabilities of 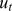 being 1 that give us a measure for the prediction of his outcome.

In this particular case the outcome refers to the pCR to the treatment of patients in a breast cancer study, which is defined as a complete disappearance of the tumor with no more than a few scattered tumor cells detected by the pathologist in the resection specimen [16].

As before we use a mixture model (equations 1 and 2) [15] to introduce a trinary latent indicator variables for the CNA state for each probe and the expression level state for each gene, and latent Gaussian variables 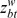 and 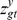 to define a probit scores for the trinary indicators 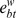 and 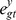 (3).

The next two equations embody our assumption that positive or negative clinical response of patients could be related to differential behaviour of a small subgroups of the 11,306 genes, i.e. copy number variation and gene expression. We assume
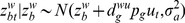
(4)where 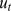 is the clinical outcome mentioned above, measured on the 116 patients, and 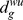 is a binary indicator introduced for controlling the number of covariate in the regression.

The integration of the two platforms is implemented as a regression with the probit scores,
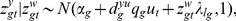
(5)where 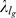 characterizes the relationship between the two platform, 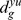 is a binary indicator introduced for controlling the number of covariate in the regression and 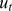 is the same variable as above.

As new patients 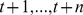 come into the study, and supposedly they do not have an information on pCR, an assumption on their outcome is made, as follows:
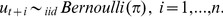
(6)


so that we can learn about 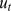 through the above prior and 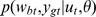, using Bayes formula and MCMC methods. The Bernoulli probability 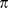 was set to be equal to the sample proportion of patients with positive pCR.

#### Priors

Priors are defined as in section 2.4, with the only exception of the regression parameters 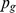 and 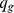, and the binary indicators 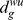 and 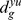. For both the first parameters an informative prior is assumed
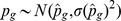


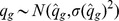
with 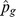, 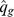 and the variances estimated using the data. While for the two indicators



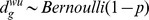


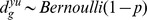
with 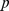 very close to 1 to allow for the selection of a very small subgroups of genes as covariates in the two regressions.

A summary of the model is given in the lower part of Figure 1.

### Bayesian Multiplicity Control

Posterior inference for the proposed model is carried out using MCMC simulations by a Gibbs sampling scheme, iterating from the complete set of full conditionals reported in the appendix.

Since the analysis deals with high throughput gene expression data and our final aim is that of selecting 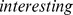 genes [17] multiple comparison problems arise.

A useful generalization of frequentist Type-I error rates to multiple hypothesis testing is the false discovery rate (FDR) introduced in Benjamini and Hochberg [18], and reviewed in a Bayesian framework by Storey [19], [20].

Let 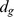 denote the indicator for gene 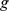 being differentially expressed under two biological conditions of interest (in our case we will be facing two different indicators 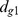 and 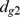 whether the comparison is ER+ vs TN or HER2+ vs TN).
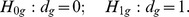



Let 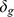 denote an indicator for rejecting the 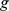-th comparison and 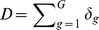 denote the number of rejections; it is defined 
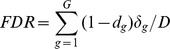
as the fraction of false rejections, relative to the total number of rejections. As such it is neither Bayesian nor frequentist. Under a Bayesian perspective, since the only unknown quantity is 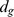 in the numerator, it can be defined an expected FDR. Let 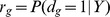, then



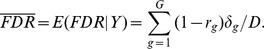



It was proved by M
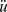
ller et al. [21] that under several loss functions that combine false negative and false discovery counts the optimal rule is of the following form 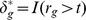. The problem is now that of specifying 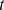 so that the FDR is controlled at a desirable level.

An algorithm that allows us to compute FDR levels for number of discoveries, and therefore to select differentially expressed genes so that the FDR level is controlled at level 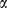, consists in sorting, from the lowest to the highest, the marginal posterior probabilities 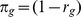, to obtain 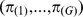. Thus, if 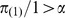, we do not reject any null hypothesis; otherwise, if 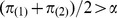, we reject 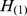 only. We iterate this procedure until the first time 
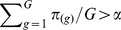
, and reject 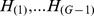.

### Simulation Study

We perform a small simulation study and generate data in a way that the last 50 (out of 1,000) genes show joint differential behaviour in copy number and RNA expression. We firstly generated two matrices for gene expression 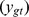 and copy number 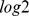 ratios 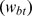, respectively of dimensions 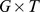 and 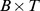, with 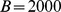 probes, 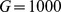 genes (exactly two probes per gene) and 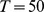 samples. The clinical covariate 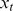 is set to be 1 for the first 10 patients and 0 for the remaining 40 patients. Sample and gene effects were generated from the corresponding priors in the model, 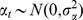 subject to 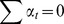 and 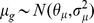. Observed log2 ratios and expression values were sampled from two Gaussian distributions, respectively centred at 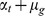 and 0. To induce differential joint behaviour for the last 50 genes, we did the following:

for RNA expression, we generated 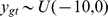 for 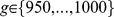 and 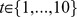;

for copy number, we generated 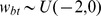 for 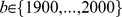 and 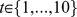;

The second simulation study generates data from the proposed mixture model. We started from setting 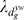 to be 2 for the first 50 genes and 0 for the remaining 950. and generated the latent scores from the corresponding priors in the model, 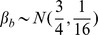 for 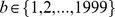, 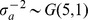, 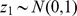 and 
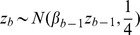
 for 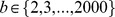, 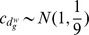 for 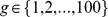 and 
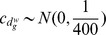
 for 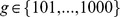, 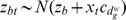 for 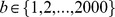 and 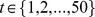, 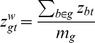, 
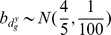
 and 
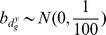
, randomly with proportions respectively 30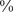 and 70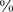, 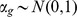 for 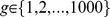 and 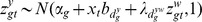. Once the latent scores are generated, using (1 and 2), we generate gene expression and CNA measurements, setting the hyperparameters as follows:
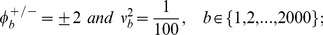


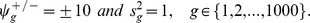



In both cases roughly 2000 iterations were needed for convergence of the MCMC chain.

**Figure 2:**
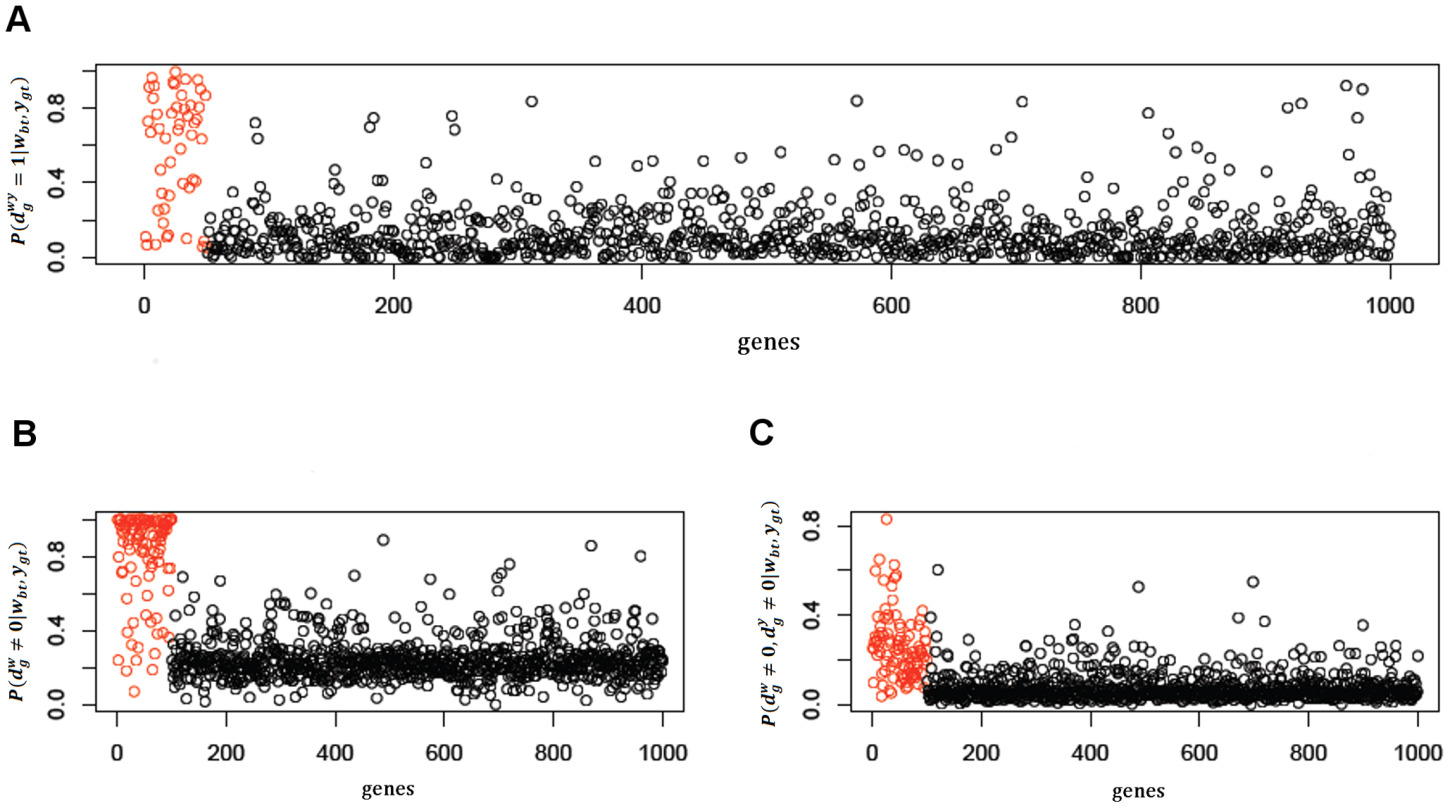
Posterior probabilities of positive interaction between the two platforms (A), differential CNA (B) and differential joint behaviour (C) after simulation 2. The red dots highlight posterior probabilities of genes which are claimed by the model to show respectively positive interaction between the two platforms, differential CNA and differential joint behaviour.

For the sake of simplicity, we report only results for the second simulation. In Figure 2 we show the posterior probabilities of positive interaction between platforms (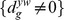), differential CNA (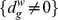) and joint CNA and RNA differential expression (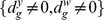). As we expected, posterior probabilities of positive interaction between platforms for the first 50 genes and posterior porbabilities of differential CNA and differential joint behaviour for the first 100 genes are among the highest.

While these simulations merely show that our proposed models achieve what is expected, we direct attention to selection of differentially behaved genes with multiplicity control and then data analysis based on breast cancer samples.

## Results

We applied our model to the breast cancer data set. As comparison, we also applied a simpler version of our models by setting 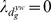 for all the genes. The simpler models assume that the gene expression and copy numbers are independent and therefore there is no integration. We call these simpler versions “marginal models”.

**Figure 3:**
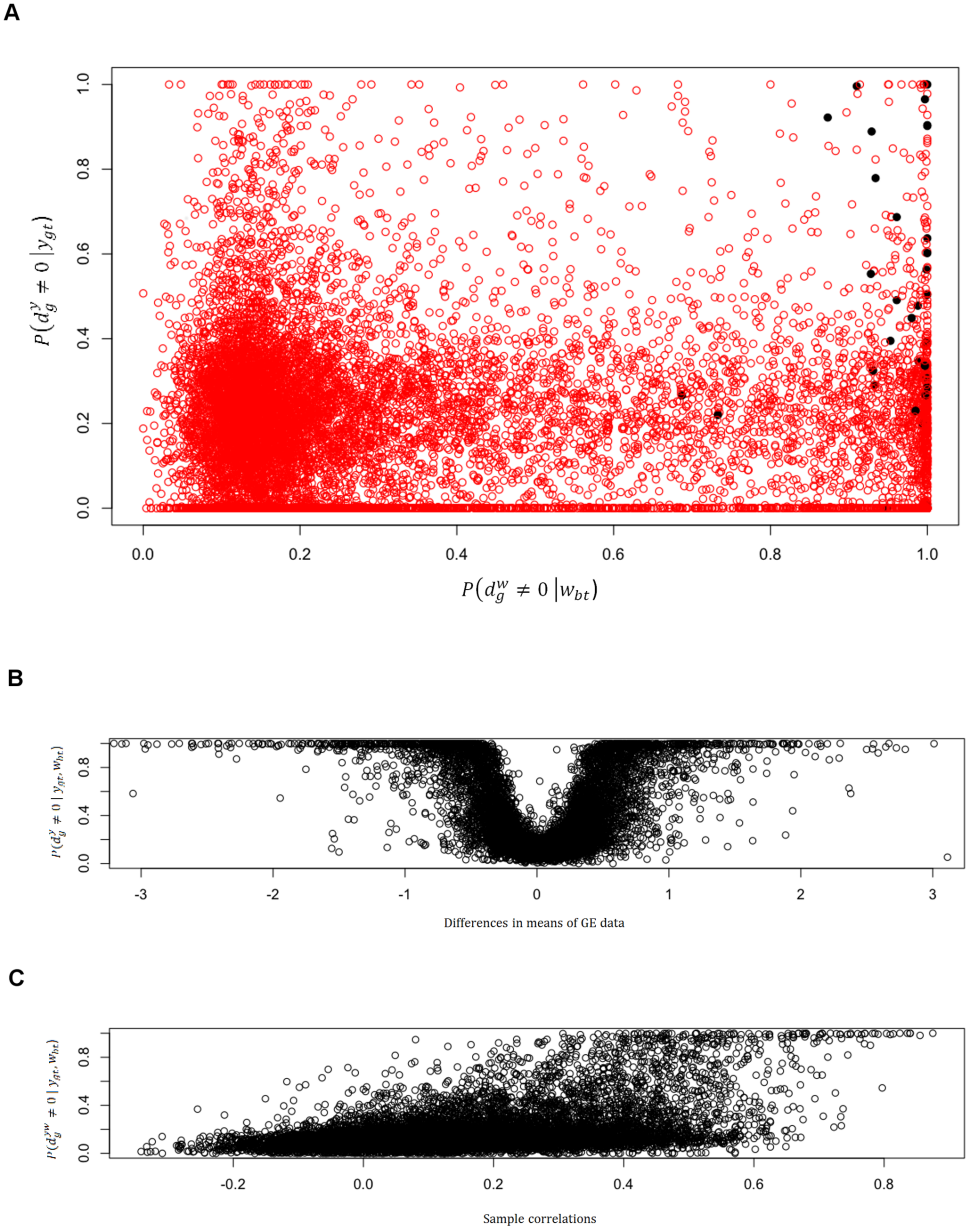
Posterior probabilities of differential CNA (on the x-axis) and differential expression (y-axis) obtained respectively through the marginal models on CNA data and gene expression data (A). Black dots highlight posterior probabilities of genes which are claimed by the model to show joint differential behaviour (A). Comparison between differences in means of the gene expression data and posterior probability of differential expression (B). Comparison between sample correlations and posterior probabilities of positive interaction between platforms (C).

In the upper plot of Figure 3 dots refer to the posterior probabilities of DNA copy number amplification, 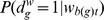, and over expression, 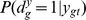, based on the marginal models; black dots highlight the list of over-expressed genes which jointly showed copy number amplification obtained through the integrated model. As expected the joint model selects, coherently, mostly genes in the upper right corner, but still differently from the intersection between the marginal ones.

A simple model checking was achieved plotting posterior probabilities of differential gene expression and difference in means of the gene expression measurements for TN and non TN group. Following the same criteria, we plotted posterior probabilities of positive interaction between platforms and sample correlations. Lower plots of Figure 3 show, respectively, a very good match between no difference in sample means and low posterior probabilities of differential expression, and between strong positive sample correlations and high posterior probabilities of positive interaction between platforms.

Our main focus was on five lists of *interesting* genes: under (over)-expressed genes which jointly showed DNA copy number deletion (amplification) in TN subgroup, under (over)-expressed genes conditional on DNA copy number aberration only in TN subgroup and genes which showed positive interaction between the two platforms. We therefore respectively defined

• 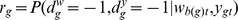



• 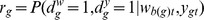



•
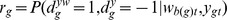



•
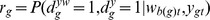



•
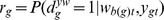
where 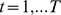 and 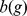 indicates all the probes belonging to the gene *g*.

FDR levels were computed with the algorithm presented in the previous section for the distinct 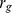
's, and genes were selected choosing a cutoff 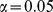 The lists of selected genes could be of greater interest for clinicians since they indicate which genes show differential expression and copy number variation in TN patients versus patients who tests positively for ER and HER2 receptors.

On the other hand, for prediction of pCR, we split the data sets into a training set and a test set; the training set, consisting of 94 patients, was used to obtain samples from the posterior distribution of the parameters while the test set, consisting of 22, to check for prediction performances through the ROC curve. Both sets were randomly selected, and numerosities with respect to pCR of training and test samples are reported in table 2. We constrained numerosities in order for the test sample to be equally balanced between positive and negative pCR, and for the training sample to respect proportions of the original data set.

**Table 2:** Numerosities in the training set and test set.

	Training sample	Test sample	TOT
Positive pCR	20	11	31
No pCR	74	11	85
TOT	94	22	116

The adopted method for the estimation of the smoothed ROC curve is LLoyd and Yong's one [22], which is proved to perform better than the empirical estimation. They proposed to estimate this curve from kernel smoothing of the distribution functions of the diagnostic measurement underlying the binary decision rule, i.e. the conditional posterior probabilities of positive pCR, and showed the significant accuracy achieved by this method for realistic sample size compared with the empirical estimation.

**Figure 4:**
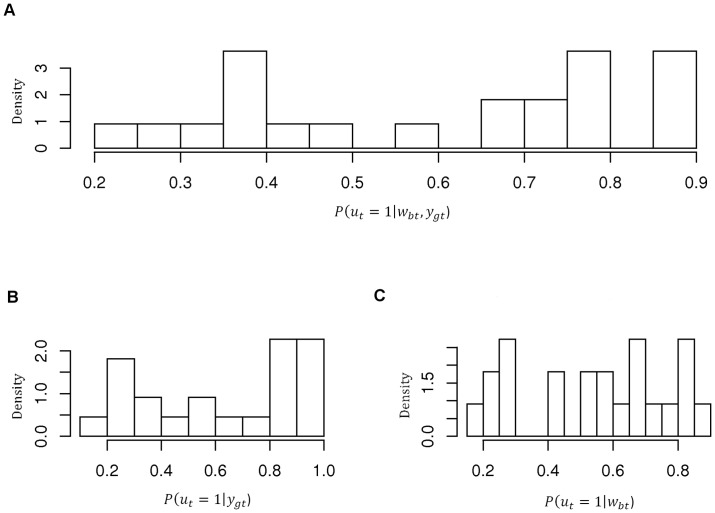
Histograms of the posterior probabilities of positive pCR in the integrated model (A) and in the marginal models, respectively on gene expression (B) and CNA data (C).

As mentioned above, the tests we performed were done on a sample of 22 patients, for which we had previously measured their pCR, and are based on the posterior probabilities of the clinical outcome being 1, 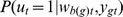, obtained running the Gibbs Sampler for 30.000 iterations. We performed the same analysis using marginally the two platforms and obtaining respectively posterior probabilities 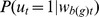 and 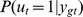. These posterior probabilities, obtained through the joint and marginal models, are showed in Figure 4.

**Figure 5:**
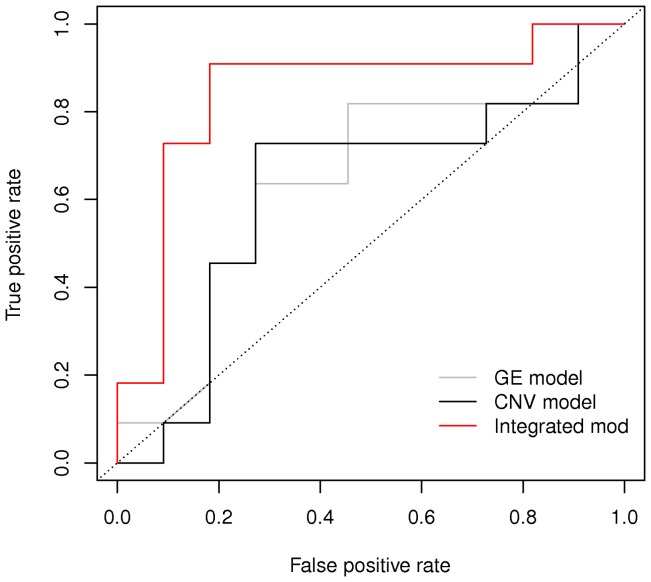
Comparison between ROC curves obtained with the marginal and integrated model.

The ROC curves are compared in Figure 5 and such comparison confirms our choice of borrowing information between the two genomic platforms, since the ROC curve corresponding to the integrated model has by far the highest Area Under the Curve, slightly below 0.9.

**Figure 6:**
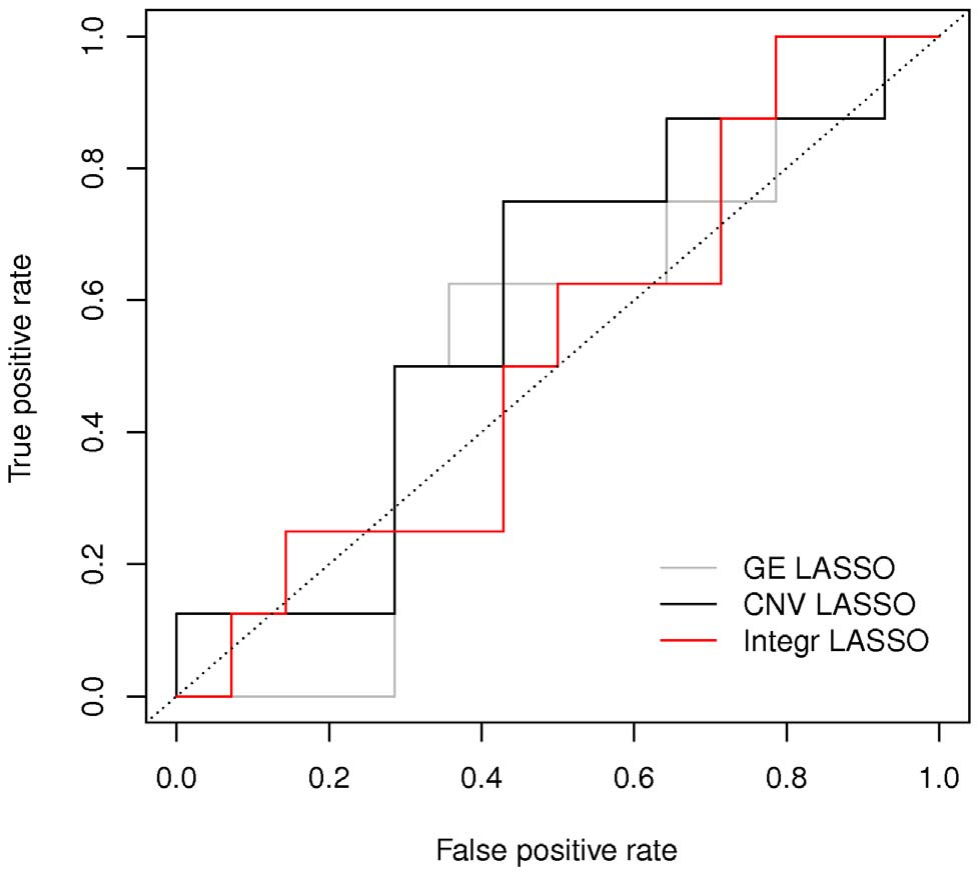
Comparison between ROC curves obtained with the LASSO logistic regression, respectively using single or joint platforms.

We finally tried and compared our method with a simple logistic regression with LASSO variable selection (LLR) [23] [24], whose corresponding ROC curves are plotted in Figure 6. We performed the analysis using the package *glmnet* in R, and set the elastic net mixing parameter 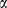 to 1. The penalty is defined as
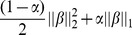
and 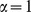 correponds to the Lasso penalty, which in this case gave the best prediction performances.

**Figure 7:**
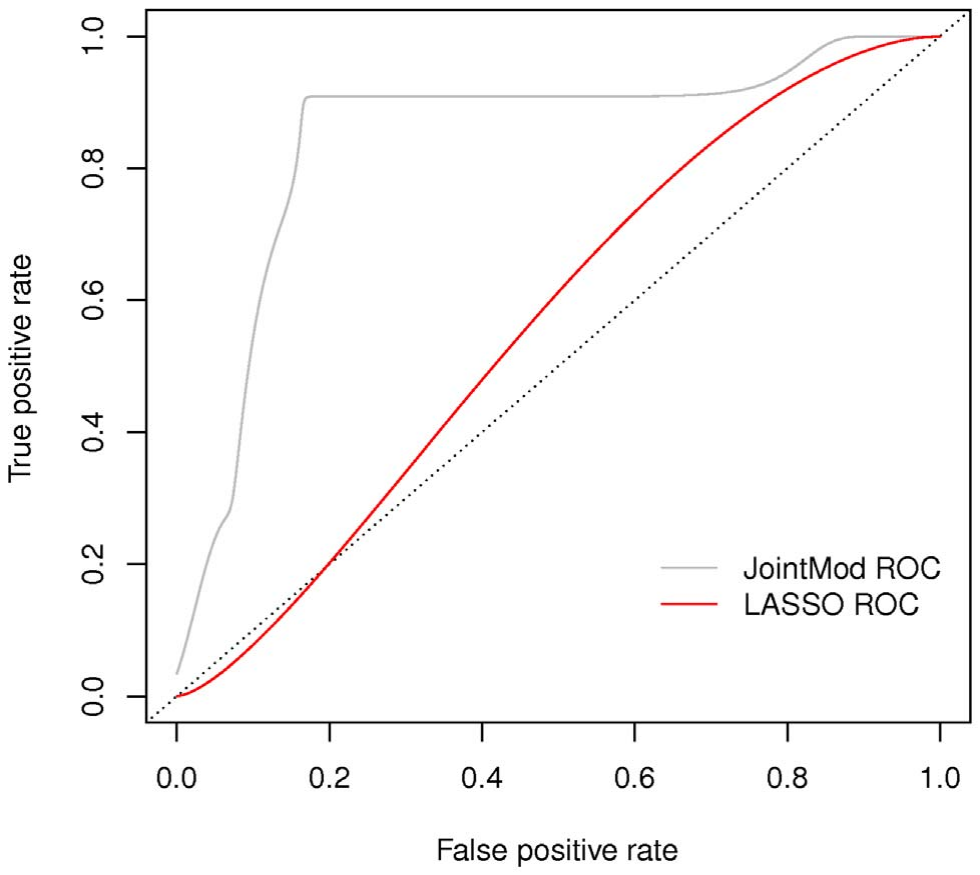
Comparison between ROC curves obtained with the integrated model and LASSO logistic regression of pCR on copy number data.

We therefore plotted in Figure 7 the smoothed ROC curves based on posterior probabilities of pCR obtained through the integrated model and on predictive probabilities obtained through LLR using only copy number variation data. The AUC under the curve obtained through our integrated model shows to be much higher that the one under the curve obtained through LLR.

## Discussion

We have introduced a Bayesian hierarchical model to integrate two types of genomics data, copy number and RNA expression. The proposed model can be easily extended to multiple platforms, with modification to the modeling of latent probit scores. Since the entire statistical inference is based on a coherent probability model, scientific questions can be addressed with probability statements, allowing for reporting uncertainty measures such as FDR. This is the main advantage of the proposed models over existing ones.

In table 3 we reported the list of genes which show jointly over expression and copy number amplification in TN patients, which was of great interest for clinicians and was also the list associated with the lowest FDR levels. Gene MYC appeared in the list and the result is promising since MYC is a key regulator of cell growth, proliferation, metabolism, differentiation, and apoptosis and MYC deregulation contributes to breast cancer development and progression and is associated with poor outcomes. Multiple mechanisms are involved in MYC deregulation in breast cancer, including gene amplification, transcriptional regulation, and mRNA and protein stabilization, which correlate with loss of tumor suppressors and activation of oncogenic pathways [25].

**Table 3:** List of genes which jointly show over expression and copy number amplification in TN group.

Symbol	EntrezID	Cytoband	postprob
E2F3	1871	6p22	0.951
MYC	4609	8q24.21	0.954
PLCG2	5336	16q24.1	0.954
PEPD	5184	19q13.11	0.954
C12orf32	83695	12p13.33	0.954
C10orf10	11067	10q11.21	0.954
FOLH1	2346	11p11.2	0.955
GTPBP2	54676	6p21	0.956
KARS	3735	16q23.1	0.957
CD14	929	5q22-q32	0.958
SHCBP1	79801	16q11.2	0.959
CHD1L	9557	1q12	0.959
CCDC86	79080	11q12.2	0.962
SLAMF7	57823	1q23.1-q24.1	0.962
CTPS	1503	1p34.1	0.962
IRAK1	3654	Xq28	0.964
C1GALT1	56913	7p14-p13	0.965
STK38	11329	6p21	0.965
AK2	204	1p34	0.966
HEPH	9843	Xq11-q12	0.966
VIM	7431	10p13	0.967
CDH3	1001	16q22.1	0.968
TRIT1	54802	1p34.2	0.969
GAS1	2619	9q21.3-q22	0.971
HLA-DRA	3122	6p21.3	0.972
ST8SIA1	6489	12p12.1-p11.2	0.973
FXYD5	53827	19q13.12	0.975
C1S	716	12p13	0.975
RECK	8434	9p13.3	0.976
C11orf75	56935	11q21	0.976
MOBKL2B	79817	9p21.2	0.977
HLA-E	3133	6p21.3	0.978
FAM107A	11170	3p21.1	0.979
ICAM1	3383	19p13.3-p13.2	0.979
INSL4	3641	9p24	0.980
PRKD3	23683	2p21	0.982
SLC2A3	6515	12p13.3	0.983
PVR	5817	19q13.2	0.984
TPX2	22974	20q11.2	0.985
NDRG1	10397	8q24.3	0.985
NFKBIE	4794	6p21.1	0.985
TIMM44	10469	19p13.3-p13.2	0.986
C1orf38	9473	1p35.3	0.986
PDSS1	23590	10p12.1	0.986
SH2D2A	9047	1q21	0.986
USP25	29761	21q11.2	0.989
HMGN4	10473	6p21.3	0.989
CHODL	140578	21q11.2	0.990
POLR1E	64425	9p13.2	0.990
STIL	6491	1p32	0.992
BTG3	10950	21q21.1	0.992
MCM4	4173	8q11.2	0.992

Breast cancer has been classified into 5 or more subtypes based on gene expression profiles, and each subtype has distinct biological features and clinical outcomes. Among these subtypes, basal-like tumor is associated with a poor prognosis and has a lack of therapeutic targets. MYC is overexpressed in the basal-like subtype and may serve as a target for this aggressive subtype of breast cancer. Tumor suppressor BRCA1 inhibits MYC's transcriptional and transforming activity [25]. Loss of BRCA1 with MYC overexpression leads to the development of breast cancer, especially, basal-like breast cancer. As a downstream effector of estrogen receptor and epidermal growth factor receptor family pathways, MYC may contribute to resistance to adjuvant therapy. Targeting MYC-regulated pathways in combination with inhibitors of other oncogenic pathways may provide a promising therapeutic strategy for breast cancer, the basal-like subtype in particular [26].

As far as the model is concerned, there are a few possible weaknesses in the procedure, mainly related to the prior specification for parameters 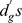, related to differential expression and prediction. We were dealing with highly parametrized models and few observations data sets, reason why we chose some easier shortcuts in order to achieve faster MCMC convergency. Some interesting modifications of our prior specifications are now to be implemented, since we found in literature new and more efficient approaches to the issue of sparsity, such as the horseshoe prior [27].

Also, it was very hard to compare our models' performances with other methods, either due to the lack of codes or to the scarcity of works on the specific topic of prediction using integrated genomic platforms; we therefore chose a simple LASSO logistic regression which showed to be a poor fit for this particular data and this is mainly due to the high correlation between them.

Future work includes the development of models for integration of three or more platforms, and the extension to new type of genomics data, such as next-generation sequencing (NGS) data. In the latter case, the main challenge is the inclusion of a model for the count data from the NGS experiment. The intuitive statistical method for such an extension would be a graphical model, where network priors will be considered treating each platform as a node, and edges among the nodes will be interpreted as dependence between platforms.

Finally, all this project was focused on a specific data set, with rather particular features. The natural hierarchical structure and correlation between DNA and RNA makes very hard to think of the application of our methodology to different problems, though an interesting path to follow could be that of demographical sciences, where this hierarchical structure could be found for example in data at country level and regional level.
